# Recent Advances in Chemical Biology Using Benzophenones and Diazirines as Radical Precursors

**DOI:** 10.3390/molecules25102285

**Published:** 2020-05-13

**Authors:** Muhammad Murtaza Hassan, Olasunkanmi O. Olaoye

**Affiliations:** 1 Department of Chemical and Physical Sciences, University of Toronto Mississauga, 3359 Mississauga Road North, Mississauga, ON L5L 1C6, Canada; tobi.olaoye@mail.utoronto.ca; 2 Department of Chemistry, University of Toronto, 80 St. George Street, Toronto, ON M5S 3H6, Canada

**Keywords:** photoaffinity labelling, benzophenone, diazirine, radical precursors, interactome, SABRE, hyperpolarizing agents, crosslinking, photochemistry

## Abstract

The use of light-activated chemical probes to study biological interactions was first discovered in the 1960s, and has since found many applications in studying diseases and gaining deeper insight into various cellular mechanisms involving protein–protein, protein–nucleic acid, protein–ligand (drug, probe), and protein–co-factor interactions, among others. This technique, often referred to as photoaffinity labelling, uses radical precursors that react almost instantaneously to yield spatial and temporal information about the nature of the interaction and the interacting partner(s). This review focuses on the recent advances in chemical biology in the use of benzophenones and diazirines, two of the most commonly known light-activatable radical precursors, with a focus on the last three years, and is intended to provide a solid understanding of their chemical and biological principles and their applications.

## 1. Introduction

**Scheme 1:**
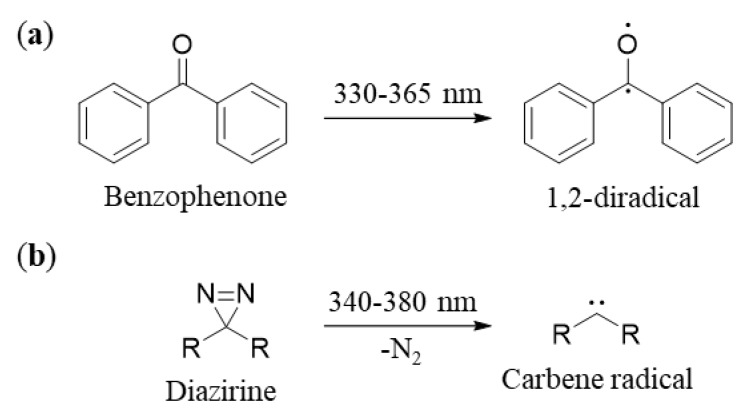
Two commonly used radical precursors in chemical biology. (**a**) Benzophenones generate a 1,2-diradical upon irradiation with 330–365 nm light. (**b**) Diazirines liberate nitrogen and generate a carbene radical upon irradiation with 340–380 nm light.

The use of radicals or photoactivatable radical precursors has become ubiquitous in the fields of medicinal chemistry and chemical biology in the past three decades. Their main application revolves around studying the interactions of biomolecule targets such as proteins with ligands containing photoactivatable handles. These photoreactive chemical groups (PCG) can be added to a probe, ideally with minimal perturbation of the intrinsic activity of the probe, which is then subsequently introduced into a biological system. After a given incubation period of the photolabelled probe in the biological system, the probe can be activated by light to reveal its biomolecular interactions, thus providing spatial and temporal information. This technique is referred to as photoaffinity labelling (PL). Such applications of chemical systems to study biological systems have transformed the chemical biology field by providing insights into protein interactomes, protein–ligand binding pocket identification, and studying the promiscuity of drugs in biological systems, enabling us to answer the fundamental “what, when, and where” questions of biological interactions. This review focuses on the chemistry of two commonly used PCGs, benzophenone and diazirines, as representatives of two classes of radical precursors (1,2-diradical and carbene radical, respectively; Scheme 1), and their applications in biological systems with an emphasis on the last three years.

## 2. Chemical Principles

### 2.1. Benzophenones

**Figure 1:**
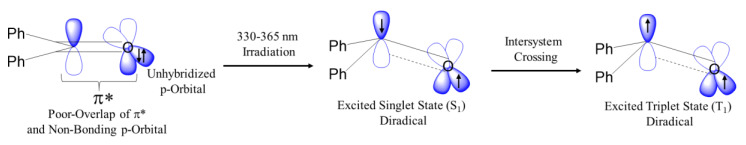
Simplified depiction of the n–π* transition; the π bond and other non-bonding lone pair in the sp-orbital of oxygen is omitted for simplicity. Excitation of a BP yields an excited singlet state (S_1_) with a distorted non-planar geometry and a longer C–O bond (bond order = 1.5), which undergoes intersystem crossing to yield the excited triplet state (T_1_).

The use of benzophenones (BPs) in PL was first recorded in 1974, when it was shown that they could efficiently label amino acids [1]. BPs, like other ketone functionalities, consist of a carbonyl carbon that undergoes intersystem crossing in high yields, making it a robust triplet photosensitizer for use in organic and biological chemistry. Its extensive chemical and physical properties have been reviewed elsewhere [2] and are not the main focus of this review; however, notable physical and chemical features pertinent to this review are discussed herein. BPs undergo two transitions, π–π*, a high-energy transition, and n–π*, a lower-energy transition. The and n–π* transition occurs at 330–365 nm and is well separated from the π–π* transition [2,3]. High-energy UV irradiation has a tendency to damage proteins due to the spectral overlap of endogenous fluorophores (e.g., tryptophan, tyrosine, histidine etc.); thus, the lower-energy n–π* transition of BPs allows them to be more applicable to biological settings [4]. Although the n–π* transition of BPs has a lower absorptive spectral overlap with biological fluorophores, it has a much weaker absorption than the π–π* transition, as indicated by its low molar absorptivity coefficient (ε_max_ < 300) [5]. This can be explained by the poor orbital overlap of the orthogonal p-orbital containing the non-bonding electron lone pair and π* orbital reducing the probability of excitation (Figure 1). The hybridization of the lone pairs on the carbonyl oxygen can be depicted in one of two ways: both lone pairs are sp^2^-hybridized, or one lone pair is sp-hybridized while the other is an unhybridized p-orbital [2,6,7]. The non-bonding orbital containing the lone pair is depicted below as an unhybridized p-orbital (Figure 1) [2,6]. The n–π* transition breaks the C=O double bond and generates a diradical, which can exist in a singlet or triplet state and is capable of efficiently crosslinking via covalent bonding with nearby chemo-moieties. The breaking of the C=O double bond lengthens the bond distance, since the promotion of one non-bonding electron to the π* orbital decreases the bond order of the C=O bond from 2 to 1.5. This also results in a change in the hybridization of the carbon atom, resulting in a change in the geometry to a non-planar sp^3^-like pyramidal structure [8].

Excitation of the BP chromophore first yields a diradical in a singlet state (S_1_), which undergoes intersystem crossing to quantitatively yield the triplet state (T_1_). The exact mechanism of the intersystem crossing is controversial [9]. El-Sayed et al. proposed a direct mechanism between the S_1_ and T_1_ excited states as indicated by the low-temperature Zeeman effect observed in spin alignment (ZESA) experiments [8]. Presumably, although the direct S_1_ to T_1_ transition violates the El-Sayed selection rules, the BP aromatic rings lose planarity upon excitation, allowing for the direct transition (Figure 1) [8]. Later studies however, showed that the process is mediated initially through intersystem crossing to a higher-energy excited triplet state (T_2_) tunable through solvent polarity, which then relaxes via internal conversion to T_1_ [9,10]. Furthermore, recent studies in the dynamics of BP triplet formation in gas-phase conditions have suggested that upon excitation to its S_2_ state, it undergoes internal conversion to S_1_ which then decays to T_1_ [11].

**Figure 2:**
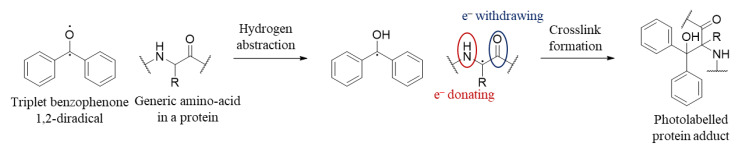
General depiction of photolabelling of protein residues (R corresponds to the amino acid side chains) by a BP. The excited triplet BP 1,2-diradical undergoes H-abstraction, leading to a stable ketyl radical and an α-carbon radical that is stabilized by the captodative effect of the electron-withdrawing carbonyl group and the electron-donating amino group. The ketyl radical and α-carbon radical recombine to form the crosslinked protein adducts.

The T_1_ state reacts as an alkoxy radical and yields an electron-deficient oxygen that reacts favorably with C–H bonds to form relatively stable carbon radicals (e.g., α-carbons of amino acids) by hydrogen abstraction (Figure 2). The resulting carbon radical formed in the hydrogen abstraction process recombines with the BP ketyl radical to form a photo-crosslinked adduct. Thus, it is the T_1_ state resulting from the n–π* transition that is mainly responsible for the formation of a covalent bond, having a quantum efficiency of ~1 for C–H abstraction and 0.1 for the π–π* transition [10]. The α-carbon radical of amino acids formed upon C–H abstraction is synergistically stabilized by captodative substituents, which allows efficient hydrogen abstraction and its subsequent labelling of virtually all amino acids (Figure 2) [12,13].

In addition to reacting with the α-carbon, the side chain functionalities of amino acids may also partake in photolabelling. For instance, the C–H bond energies for a series of stabilized radicals formed by amino acid side chains (tertiary, benzylic, and α radicals to the heteroatoms or carbonyl groups) allows for the potential labelling of all amino acid side chains through hydrogen abstraction reactions [4]. The 1,2-diradical reacts preferentially with C–H bonds over water, which affords it a higher photo-linking efficiency compared to other photo-crosslinkers such as diazirines [14,15].

BP derivatives can be red-shifted by electron-withdrawing appendages due to the lowering of their π* orbital energy. The chemistry of BPs is also sufficiently advanced that incorporation into chemical and biological probes is easy to achieve, and their inertness to solvents makes them an attractive class of PCGs for PL. Some of the many advantages that allow widespread use of BPs in chemical biology include: low spectral overlap of the n–π* (330–365 nm) with endogenous chromophores (<300 nm), quantitative quantum yields of intersystem crossing to yield the crosslinking relevant excited state, quantitative quantum efficiency of C–H abstraction of the excited state, high metabolic stability, and relative stability of the BP and its excited state [3,5]. However, they require long irradiation periods, which may result in non-specific crosslink formation and cell damage. Furthermore, their mass (182 Da) and high lipophilicity may limit their use in biological systems. Addition of large photolabels such as BPs may significantly alter the mode of interaction being studied due to added steric bulk and the ability of its π-system to interact with other aromatic amino acids [16]. The lipophilicity of molecules being studied is a main factor in biological systems, where most studied interactions depend upon the cellular permeability of the compound by passive diffusion. Furthermore, proteins are folded such that hydrophobic amino acids are less water-exposed than hydrophilic; thus, the addition of a lipophilic BP moiety to a biological probe may alter the site of interaction on a protein [16]. Nonetheless, BPs continue to be used as a popular PCGs for PL despite their drawbacks, and despite the discovery of many other PCGs since the advent of BPs [17,18].

Their application extends beyond the common PL techniques of binding-site and biological-interactome mapping, target identification and proteome profiling, and surface conjugation techniques amongst others. They are also found in natural products that have broad-spectrum biological effects such as anticancer, antiviral, antimicrobial, and anti-inflammatory effects [2,19,20,21,22]. The phototoxicity of this class of bioactive natural products is a probable adverse effect that may limit the widespread utilization of this pharmacophore in drug design [23]. Some research groups [24,25,26,27] nonetheless continue to explore BP groups in their drug discovery efforts, and the interested reader may consult a recent review [28] for further insight into the medicinal properties of BP-containing natural products. The use of BPs in PL techniques, including their design and utility in biological investigations, is discussed in Section 3.

### 2.2. Diazirines

#### 2.2.1. Chemical and Physical Properties

Diazirines (DAZs) were first discovered in 1960 upon the oxidation of diaziridines, and are quite stable unless exposed to light (~360 nm), heat, or ultrasonication [29,30,31]. DAZs are used as carbene precursors that liberate molecular nitrogen and a carbene upon longwave irradiation (~360 nm). Upon irradiation, DAZs can generate a carbene by liberating nitrogen directly, or by rearrangement to yield a diazoalkane which liberates nitrogen, thereby forming a carbene indirectly [32,33,34]. When aliphatic DAZs were first discovered, their utility was limited due to their tendency to undergo 1,2-H shifts [33,34]. Initial efforts in the 1960s aimed at using carbene precursors such as diazoacetates for PL [35] which were later superseded by phenyldiazirines and spiro-adamantane-2,2′-diazirine, as introduced by Bayley and Knowles in 1978 [36]. This promising discovery allowed the use of photoactivated carbenes at long wavelengths that biological groups such as amino acids and DNA do not absorb, but, however, suffered from the rearrangement to a linear diazoisomer, which is highly sensitive to acids and nucleophiles [33]. Subsequently, Brunner et al. invented a 3-trifluoromethyl-3-phenyldiazirine (TPD) group that was resistant to such hydrogen rearrangements and reduced the yield and reactivity of the diazoisomer, thereby increasing its applicability for PL and exhibiting nearly quantitative (>95%) OH insertion yields in methanol [33].

**Figure 3:**
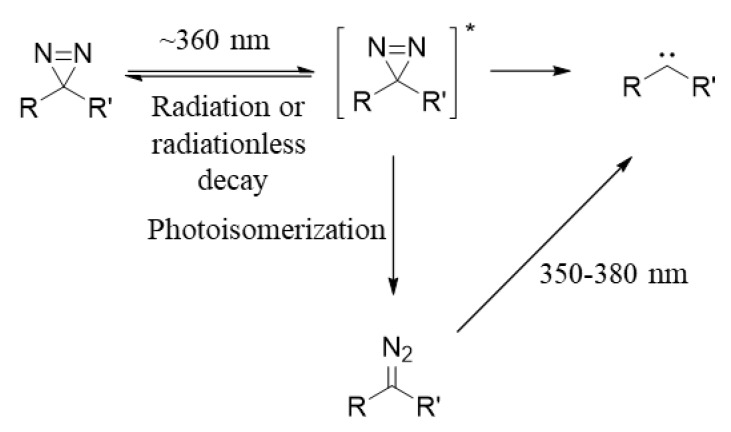
Possible relaxation pathways of excited DAZs. Upon excitation of DAZs by long-wave UV irradiation, they can relax by internal conversion, isomerize to the diazoisomer, or form a carbene directly.

**Figure 4:**
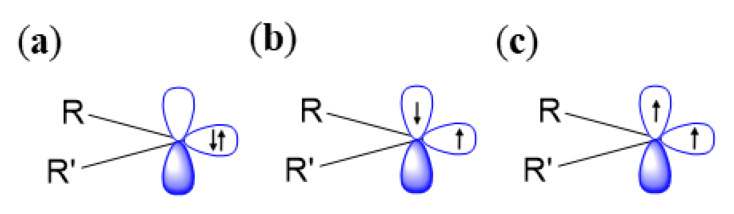
Possible open/shell states and spin multiplicities of carbenes formed from DAZs. (**a**) Closed-shell singlet carbene. (**b**) Open-shell singlet carbene. (**c**) Triplet carbene, usually generated by the addition of a photosensitizer.

Upon excitation of DAZs to the S_1_ state, they can decay via four main pathways (Figure 3 and Figure 4): (1) internal conversion to the singlet ground state (S_0_, Figure 3); (2) liberation of nitrogen and formation of closed-shell singlet carbene (Figure 3 and Figure 4a); (3) formation of open-shell singlet carbene (Figure 3 and Figure 4b); and (4) internal rearrangement to the diazoisomer (Figure 3) [37].

Carbenes can exist in three possible states (Figure 4). Presumably, carbenes resulting from homolytic cleavage are triplet carbenes (two unpaired electrons, each occupying a different orbital), whereas a heterolytic cleavage results in a singlet carbene (two paired electrons in same orbital), even though the triplet carbene is lower in energy (Figure 4c). The carbenes generated from DAZs, like other carbenes, are usually closed-shell singlet carbenes (Figure 4a) unless a photosensitizer is used to promote intersystem crossing to the triplet state (Figure 4c) [34,38].

Carbene spin-states can be influenced by substituents and solvent effects [39]. For instance, Sheridan’s group showed that 2-benzothienyl(CF_3_)carbene is a ground-state triplet, whereas the Platz’s group showed that in polar solvents, the 4,4′-biphenyl(CF_3_) carbene has a singlet ground-state and the singlet-to-triplet intersystem crossing rate depends on the coordinative capability of solvents [39,40]. Computational density functional studies (DFT) studies (B3LYP/6-31+G(d) and RI-CC2/TZVP; singlet excited singlet states (S_1_, S_2_, and S_3_) optimized with RI-CC2/TZVP) of phenydiazirine predicted the excited singlet state (S_1_) to be as a result of an s–p* transition [37,41].

**Figure 5:**
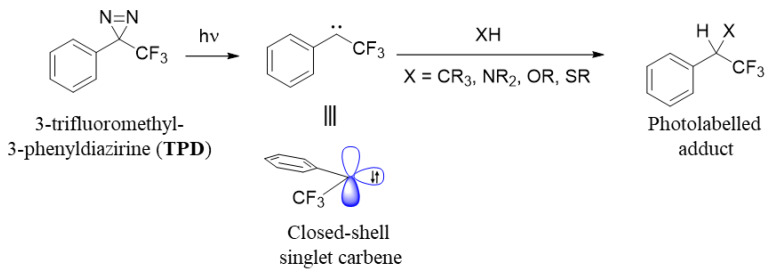
Photolabelling of proteins by 3-trifluoromethyl-3-phenyldiazirine (TPD) a commonly used DAZ motif. Irradiation of TPD generates a closed-shell singlet carbene which carries out an insertion into polar X–H bonds to give the photolabelled covalent adduct.

The reactivity of a singlet carbene differs greatly from that of a triplet carbene. A triplet carbene can be detected via electron paramagnetic resonance (EPR) and reacts as a geminate diradical that can undergo H-abstraction or be oxidized by molecular oxygen to a ketone, but is incapable of inserting into an O–H bond [42,43]. A singlet carbene reacts with insertion into X–H bonds (X = CR_3_, NR_2_, OR, or SR) to form photo-linked adducts, making the singlet state more relevant for PL (Figure 5) [44,45]. Although a singlet carbene can be either an open- or a closed-shell species, the photo-crosslinking is most likely due to the closed-shell singlet species, since the open-shell singlet, much like the triplet carbene, acts as a biradical, whereas the closed-shell singlet owes its reactivity to a zwitterionic character caused by one fully occupied orbital and another empty orbital (Figure 5) [46,47].

It may be worthwhile to note, however, that the exact mechanism for the formation of the closed-shell carbene was speculated by Platz and co-workers to be formed through the open-shell singlet intermediate [37].

#### 2.2.2. Recent Synthetic Developments

Carbenes generated from DAZs have a tendency to be quenched by water, thereby lowering their crosslinking yields (>10%) [29,43]. This is both a limitation and an advantage, since it ensures that all photo-crosslinked targets are those in immediate proximity to the DAZ at the instance of irradiation, as the carbene generated by photoactivation is too short-lived (pico- to nanoseconds) for off-target activity [29,48].

The utility of DAZs as probes for covalently mapping transient molecular interactions has prompted synthetic chemists to create easier, safer, and more efficient methods for DAZ incorporation into biological probes. Synthetic developments and reaction compatibility of DAZs have been reviewed in detail previously [29,45,49] and are not the focus of this review; brief mention is made of recent improvements and developments that may aid the reader.

**Figure 6:**
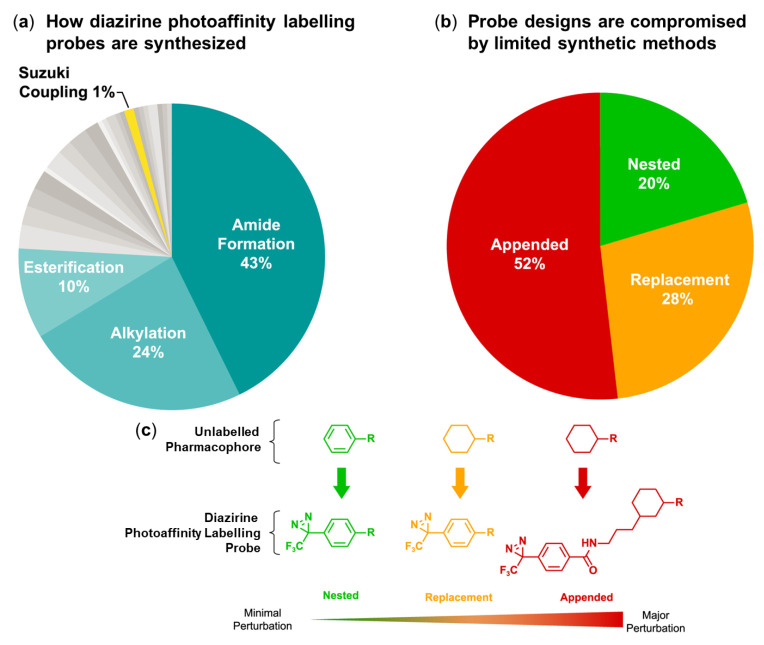
The method of synthesis and mode of incorporation of DAZ probes based on a survey of literature, including 212 published DAZ photolabelled probes. (**a**) The percentage distribution of common synthetic methods of attachment of the DAZ containing moiety to the probe. (**b**) The percentage distribution of the three categories of incorporation of DAZs (appended, replacement, nested). (**c**) A schematic representation of the three categories of DAZ incorporation and the corresponding level of perturbation caused (adapted from Ichiishi et al., 2019 [50]).

In the synthesis of probes incorporating TPD or aliphatic DAZ motifs, the PCGs are usually installed near the end of the synthesis in order to prevent exposure to harsh conditions (reagents, acid, heat, microwaves, etc.) [50]. Chemical biologists and medicinal chemists are thus constrained in terms of when and where PCGs can be installed during synthesis. The incorporation of DAZs can be categorized into three main categories: (1) appended, (2) replacement, and (3) nested (Figure 6b). An ideal photolabelled probe should be structurally and physicochemically similar to the unlabelled probe in order to provide minimal perturbation to its pharmacodynamic (potency, interactome, etc.) and physical (lipophilicity, pK_a_, ionization, etc.) properties. Nested DAZs provide minimal perturbation and replacement offers a slight perturbation, whereas appended DAZs provide the greatest degree of perturbation (Figure 6c) [29,50]. Imaginably, the greater the perturbation to the probe (such as for appended and replacement DAZs), the less representative a study using the labelled probe may of the unlabelled probe.

Because of the thermal stability and incompatibility of DAZs with metallic reagents, the current synthesis routes mainly (approximately three-quarters) revolve around the use of robust and simple methods such as amide formation, alkylation, and esterification (Figure 6a). Perhaps this synthetic limitation is the reason why more than half of the DAZ-based chemical probes described to date, consist of appended DAZs (the category with the greatest degree of perturbation) (Figure 6c).

**Scheme 2:**
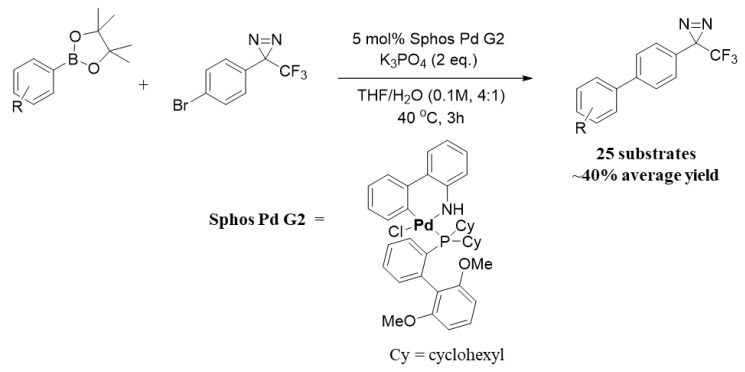
Optimized Suzuki–Miyaura reaction that is compatible with DAZs and a variety of functionalities (heterocycles, aromatic amines, alcohols, amides, etc.) and requires a short reaction time and mild reaction conditions.

In 2019 Merck and Co. published a study addressing this limitation in probe design by developing a Suzuki–Miyaura cross-coupling reaction that is compatible with TPD [50]. Suzuki–Miyaura cross-coupling screens of 8-haloquinolines with phenylboronic acid and boronic ester derivatives combined with varying reaction conditions such as temperature, palladium catalyst, solvents, and bases, in the presence of DAZ-containing spectator molecules, were used to determine the most DAZ-friendly cross-coupling conditions that gave the highest yields. After the identification of the most suitable cross-coupling conditions (least harmful to DAZs and producing the greatest yields), the substrate scope of the reaction of a TPD derivative (DAZ-containing aryl bromide (3-(4-bromophenyl)-3-(trifluoromethyl)-3H-diazirine)) was evaluated with a variety of boronate esters. The results concluded that the average yield was ~40% and that the conditions were compatible with saturated and unsaturated heterocyclic rings and hydrogen bond donors (aromatic amines, alcohols, and amides, Scheme 2). Furthermore, the applicability of cross-coupling conditions beyond the *p*-bromophenyldiazirine synthon was demonstrated by reproducing similar yields with the *m*-bromophenyldiazirine synthon. Lastly, an improvement in yield (from 20% to 45%) and reaction time (6 days to 3 h) was reported in the synthesis of known photoaffinity probe GSK-3 [50,51].

**Scheme 3:**
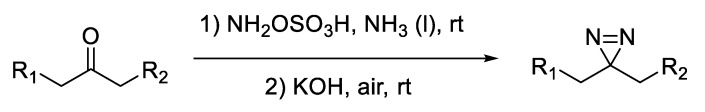
Improved one-pot, two-step synthesis of aliphatic DAZs from aliphatic ketones.

Furthermore, Wang et al. recently reported the improved synthesis of aliphatic DAZs from aliphatic ketones by using hydroxylamine-O-sulfonic acid (HOSA) in liquid ammonia for 12 h at room temperature, followed by treatment with potassium hydroxide (Scheme 3). This method produced a major improvement in yield (~30–40% to 67%) and simplicity (three steps to one pot, two steps; room temperature; under air) allowing for gram-scale DAZ production [29,52].

**Scheme 4:**
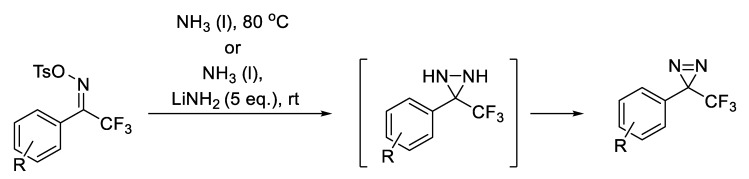
Condensed method for the one-pot synthesis of trifluoromethylphenyl **DAZ**s without isolating the diaziridine intermediate.

Additionally, Wang et al. developed two methods for synthesizing TPD derivatives in a one-pot synthesis directly from the tosyl oxime without isolating the diaziridine precursor (Scheme 4). Kinetic NMR experiments suggested the reaction to proceed via a diaziridine intermediate which is rapidly formed in situ, and is followed by a gradual oxidation to the diazirine [53].

**Scheme 5:**
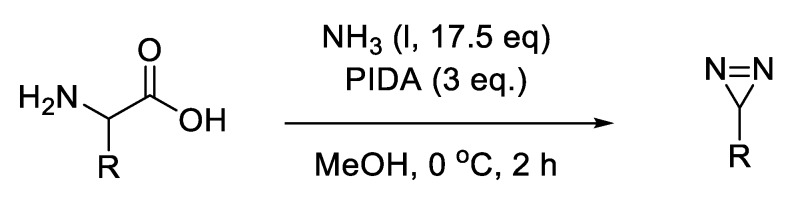
Optimized reaction conditions for the one-pot three-step diazirination reaction.

Another one-pot metal-free synthesis of terminal DAZs from unprotected amino acids using ammonia and phenyliodonium diacetate (PIDA) was developed by Glachet et al. (Scheme 5) [54]. This convenient and robust method tolerates a wide range of natural amino acids and can be scaled up to multigram quantities. The reaction proceeds via three successive one-pot reactions: (1) decarboxylation of the amino acid C-terminal, (2) formation of diaziridine upon insertion of iodonitrene, and (3) oxidation to form the diazirine. Surprisingly, this reaction was also successful in diazirination of two dipeptides by replacing its C-terminal with a terminal DAZ without racemization of the chiral centers [54], suggesting the possibility of it being applicable to polypeptides.

## 3. Applications of Benzophenone and Diazirine Radical Precursors to Chemical Biology

### 3.1. Benzophenones

#### 3.1.1. Understanding Biological Interactions and Mechanisms

**Figure 7:**
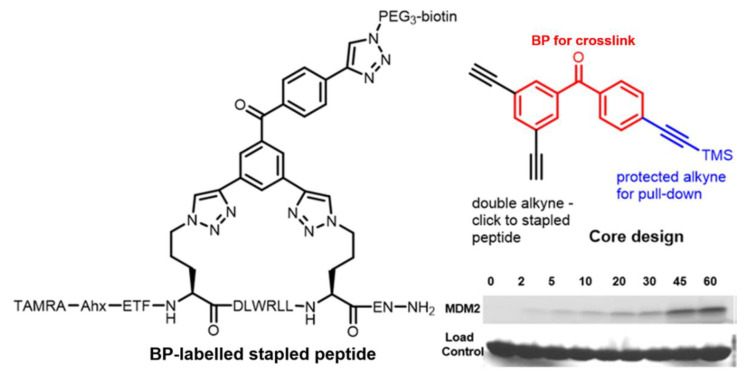
BP multifunctional linker designed and incorporated into a stapled peptide and useful in studying protein interactions with biomolecules (polyethylene glycol is abbreviated as PEG; Sodium dodecyl sulfate polyacrylamide gel electrophoresis (SDS-PAGE) adapted from Wu et al., 2016 [56]).

PL has become an important technique in elucidating how drugs, proteins, and biological molecules interact by identifying novel mechanisms and targets of therapeutic value. BPs have found extensive application in PL studies of complex biological systems such as protein–protein interactions (PPIs) [55]. Wu et al. [56] reported a BP-based multifunctional probe that incorporated two alkyne handles for double-click chemistry to create a stapled peptide and a third alkyne handle for target enrichment and pulldown (Figure 7, left). Stapled peptides are mostly hydrocarbon-braced peptides locked into their α-helixes for stabilization, permeability, improved activity, and protease resistance [57,58,59]. This strategy has been shown to enable very efficient biological targeting and investigation.

In this study, the probe was attached to the peptide via Cu-catalyzed click chemistry using the double alkyne moieties, to crosslink two different amino-acid residues of the peptide and create an α-helix stabilized by the BP-containing synthetic brace (commonly referred to as stapling). Subsequent irradiation at 365 nm allowed for crosslinking to interacting proteins in proximity, giving insight into protein-interacting partners of the peptide (Figure 7). Following crosslinking, the third alkyne handle allowed subsequent Cu-mediated click chemistry for sample enrichment with biotin. Subsequently, pulldown and proteomic mass spectrometry (MS) analysis was used to identify the target proteins.

This design was adopted into a p53-derived peptide probe and was effective in crosslinking mouse double minute 2 homolog (MDM2), a negative regulator of the p53 tumor suppressor, thus validating the efficiency of the designed probe. The time-dependent labelling of MDM2 was also observed via SDS-PAGE (Figure 7). The simple design of these BP probes allows for their integration into several study designs. The core scaffolds are easily amenable templates that can be used for a broad range of applications.

Because BPs are a feature of some natural compounds, sometimes only minimal modifications may be required for their use in PL studies, which ensures that their innate activity is unperturbed, and the results obtained can be trusted to be reliable. Zhao et al. [60] used elegaphenone (ELP), a naturally occurring antibiotic for activity-based protein profiling (ABPP), which helped to elucidate ELP’s binding partners and hence its cellular pathway in Gram-positive and Gram-negative bacteria. They incubated the natural compound with bacterial cells, irradiated cells at 365 nm, lysed, and performed a biotin–azide click conjugation (facilitated by an alkyne tag initially introduced to the *para* position to the BP in ELP) for pulldown and isolation of crosslinked targets. Subsequent LC-MS/MS analysis afforded complete proteomic studies that validated already known targets and suggested novel ones of ELP in its antibiotic mode of action.

**Figure 8:**
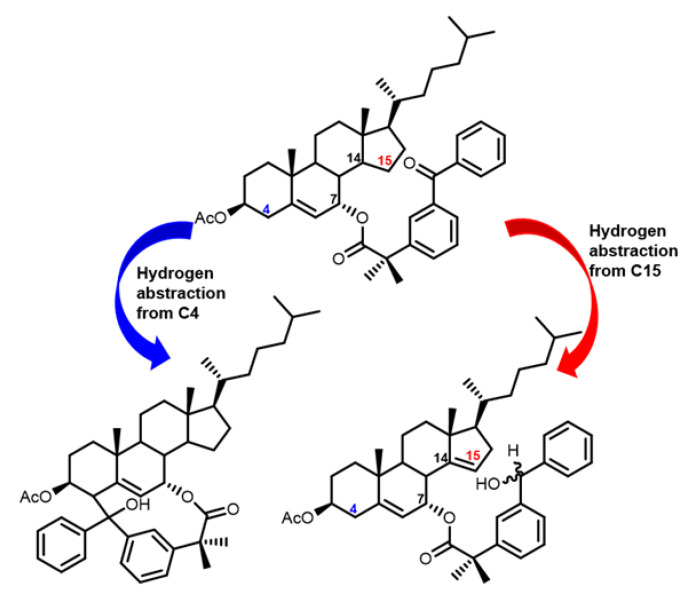
BP-modified cholesterol was used to identify possible regions of hydrogen abstraction in the cellular oxidation mechanism of cholesterol.

Uncovering the sites that confer activity and mediate interactions in a biomolecule is one way of proposing its mechanism of action. The oxidation of cholesterol is linked to several cancers and pathologies, and it is believed to proceed via intramolecular hydrogen abstraction mediated by distinct enzymatic and free-radical pathways. The use of BP in hydrogen abstraction has already been reported [61,62], and hence may be easily applied to understanding possible sites of hydrogen abstraction in cholesterol. Palumbo et al. [63] thus modified cholesterol with a BP at the C7 position and investigated possible sites of hydrogen abstraction following photoirradiation. While confirming the already known location of hydrogen extraction at C4, they also observed a similar occurrence at C15, a location that is thermodynamically unfavorable (Figure 8). The authors suggested that based on their finding, the activity and mechanism of cholesterol may be highly dependent on the specific reactant topology, and that this finding may be of interest in studying the reactivity of surface membrane.

The signaling mechanisms that trigger host responses at the cellular level are sometimes vaguely understood, and, in the case of hormonally controlled virulence of pathogenic bacteria, a BP-based probe was used to reveal cellular interactions involved in this process [64]. The peptide hormone dynorphin-A (Dyn-A) is known to be involved in cellular responses to pain and stress via enhanced virulence of the pathogenic bacteria, *Pseudomonas aeruginosa* (PA), which is involved in cystic fibrosis and may be associated with lung infections. The challenge in making treatments for this pathogen is in its elevated resistance to antibacterial therapies, and a poor understanding of its cellular pathways. Wright et al. [64] used mimics of Dyn-A that involved a BP PCG and an alkyne tag to discover Dyn-A-associated proteins in PA. They incubated their probe with the bacterial cell lysate, conducted UV-mediated crosslinking, and analyzed the pulled-down samples by LC-MS/MS. The authors subsequently took the top hit from this study, a membrane sensor kinase known as Pars, and through mutation studies were able to validate its role in PA’s cellular mode of action.

**Figure 9:**
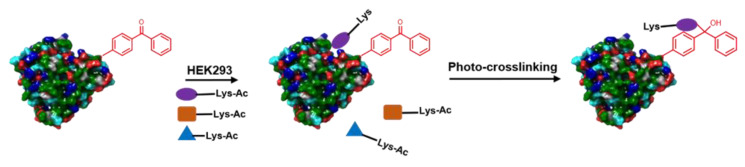
Mutant HDAC8 bearing BP-modified amino acids that can be used to identify novel HDAC8 substrates.

Substrate identification for enzymes provides key information on their cellular roles and mechanisms. PL approaches are one of the most widely applied techniques for identifying and validating new substrates for enzymes. Histone deacetylases are a protein family that enzymatically deacetylate lysine residues mediating a cascade of cellular remodeling that ends in suppression of gene transcription [65,66]. Histones were initially thought to be the only substrates HDACs act on, but a series of proteomic studies have elucidated and validated other HDAC substrates. Lopez et al. [67] were able to use a BP-mediated crosslinking technique to map proteomic interactions of one of the clinically relevant HDACs, HDAC8, in cell lysate, and identified an unbiased subset of substrates. Site-directed mutagenesis was used to incorporate BP at I94, Y100, and F191 in the HDAC8 plasmid. The plasmid was expressed using bacterial culture to obtain the mutant HDAC8, which was then subjected to an HDAC8 activity assay which validated that the mutant enzyme’s catalytic property was intact [68]. This mutant HDAC8 was then incubated in HEK293 cell lysate and UV-irradiated for 30 mins to probe for its interacting partners. The crosslinked samples were enriched and extracted using an affinity column, and then analyzed by MS to reveal crosslinked partners (Figure 9). This approach is simple and could reasonably be adapted to several proteins and biomolecules of choice.

#### 3.1.2. Other Applications Incorporating BP Probes

**Figure 10:**
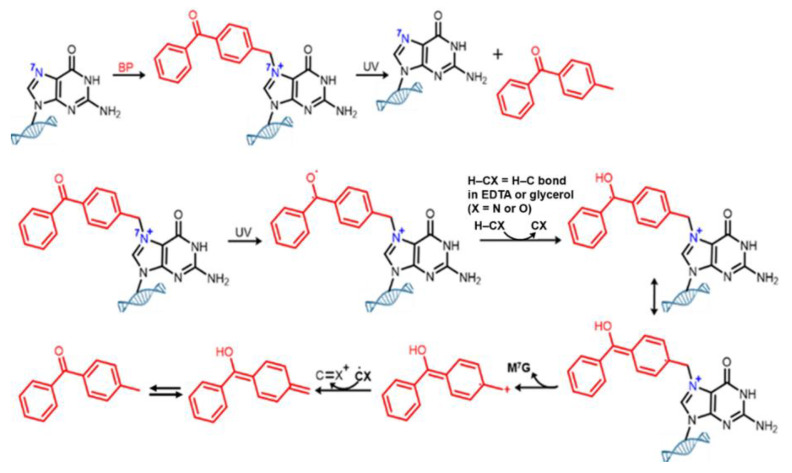
A BP-based photo-caging approach was used to study DNA interactions mediated by the m^7^G modification.

PL techniques based on BP-mediated photoactivation formation remain attractive due to their easy incorporation, minimal effects on biological processes, simple and non-invasive designs, and the ability to control their timing and location of activation which makes them suitable for a host of studies. As such, BPs have been applied to studies outside the realm of protein profiling. Photo-caging, like protecting groups, is one such application involving the masking of an interacting site on a biomolecule such that time-specific irradiation cleaves the PCG and allows restoration of function and activity [69]. Methylation of C7 on guanine (m^7^G) is the most observed nucleoside modification, mediating the functions and interactions of several nucleic acids, and understanding the molecular interactions that require this modification is highly desirable. Anhäuser et al. [70] recently reported a photocaged m^7^G using BP and they observed light-controlled uncaging, a feature that has the potential to facilitate new studies of molecular interactions of m^7^G and m^1^A (methylated adenine) and could enable the control of biological functions using light [70,71]. They proposed an uncaging mechanism that is driven by the protonated N7 and involves the biradical BP species abstracting hydrogen and transferring a lone radical to nearby chemical groups (EDTA and glycerol were used in this study) to form a trivalent carbon radical, which subsequently tautomerizes, is terminated, and regains aromaticity to form the methyl BP product (Figure 10). This product was proposed based on NMR and UHPLC-MS/MS analysis of the byproducts following irradiation.

In a dual crosslinking and immobilization approach, Jakubovska et al. [72] functionalized N^4^-cytidines with BP and demonstrated the dual usefulness of the installed BP. Through BP-mediated crosslinking, they successfully (1) immobilized the modified oligonucleotides to polymeric solid supports and (2) covalently labelled interacting proteins of the oligonucleotide. The single-stranded DNA was also able to efficiently recognize and hybridize with its complementary sequence, further expanding the usefulness of this approach. This dual approach presents opportunity for reduced functionalization and retention of innate activity in oligonucleotide studies, as BP-modified cytidine nucleobases are efficiently read and incorporated by several DNA polymerases, and the terminal deoxynucleotide transferase, TdT, is able to generate multi-BP-modified single-stranded DNA oligonucleotides.

These studies continue to highlight the utility and widespread applications of BP PCGs as a versatile tool in chemical biology.

### 3.2. Diazirines in Chemical Biology

Diazirines (DAZ) are perhaps the smallest of all the PCGs, and this affords them the added advantage of nominal alteration of the properties of the probe into which they have been introduced. This singular feature distinguishes DAZs from other PCGs and has made them very attractive for use in the study of biological processes and interactions. Upon photoactivation, DAZ generates a transient divalent carbene intermediate that is highly reactive and unstable, and rapidly interacts through covalent bond formation with nearby chemical species. This super-reactive feature of this carbene intermediate is double-edged. On one hand, this affords DAZs high selectivity and reduces their propensity to bind non-specifically, because they quickly react with proximal co-interacting biomolecules. This also minimizes the probability that they will react with any random diffusing species. Because irradiation occurs at a longer wavelength of 365 nm, and the reactive carbene intermediate generated has a lifetime of picoseconds [37,47], these species also cause the least damage to tissues and biological systems under study. However, they are very unstable and may not be suitable for long-term studies. They are also easily quenched by water, ensuring that their crosslink yields are very low [43,73]. Trifluoromethyl diazirines such as TPD produce the carbene insertion products in high yields and do not suffer from the internal rearrangement observed with non-fluorinated DAZs [33]. In studying biological systems, there is usually a matrix in which isolation of crosslinked target molecules is desired, so purification usually involves some rounds of target enrichment and then elution and analysis. Some of these designs and applications are discussed below.

#### 3.2.1. Target Engagement and Discovery of Novel Bioactive Compounds

**Figure 11:**
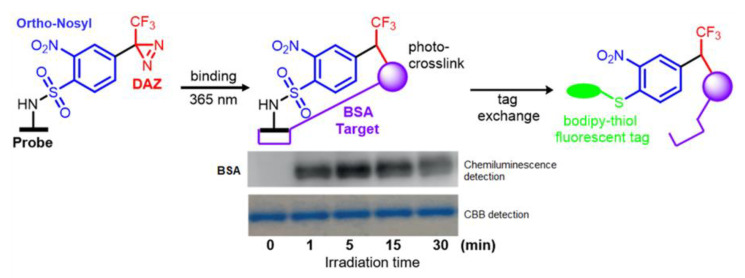
Design of *ortho*-Nosyl probe that incorporates a DAZ for use as ligand-tag exchangeable photoaffinity probe (Coomassie brilliant blue is abbreviated as CBB;gel image adapted from Saaidin et al., 2019 [73]).

DAZs have been widely used as probes for validating target engagement during the drug discovery process. A simple approach was designed by Saaidin et al. [73] that incorporated CF_3_-diazirine *meta* to the nitro group on a 2-nitrobenzosulfonamide (*ortho*-Nosyl) moiety to validate its target. Nosyl groups are excellent protective groups for amines because their deprotection is obtained in high yields following treatment with nucleophilic thiols in an S_N_Ar reaction. The approach in this study is thus useful for elucidating a drug candidate interactome, as most feature secondary amines that can afford easy installation of this PCG. Saaidin et al. installed this *ortho*-Nosyl-DAZ probe onto biotin for target validation, using bovine serum albumin (BSA) protein, a known biotin target, as an example. The biotin probe was incubated with BSA, and photoirradiation with 365 nm light (250 W) allowed detection of the BSA photoproduct via SDS-PAGE chemiluminescence method (Figure 11). Further treatment of this photolabelled BSA with bodipy-thiol allowed over 70% cleavage of the model compound via an S_N_Ar approach, and this allowed enrichment of photolabelled complexes for easier detection and identification. The authors claim that this method may be a simple approach in PL procedures that can be used to identify a probe’s interactome. This was a proof-of-concept approach which only highlighted the utility of DAZ-mediated crosslinking approaches to determine biological targets.

Similarly, a TPD-based photoaffinity probe, TPD-950-Br, was used to analyze the molecular interactions of MCC950 (also known as CP-456773 and CRID3), a known inhibitor of NLRP3 (NOD-, LRR-, and pyrin domain-containing protein 3) [74]. This study highlighted the use of a replacement-DAZ approach to generate a minimally perturbed photoaffinity probe of MCC950, which could enable future studies investigating its molecular target and mechanism of action.

Soluble guanylyl cyclase (sGC) is a nitric oxide (NO) receptor that is severely implicated in cardiovascular diseases. Novel stimulators of this receptor show a lot of clinical promise, yet their precise modes of action are largely unknown. Using a DAZ-mediated diagnostic application, Wales and coworkers showed the precise location where stimulators interact with the sGC receptor [75]. The authors used a known stimulator, IWP-051, and functionalized it with a DAZ moiety, which ensured its irreversible binding affinity to sGC upon photoirradiation. They were able to localize stimulators binding to a highly conserved β1 heme domain in human and insect sGC. Through DAZ covalent crosslinking interaction, they were also able to replicate similar localized stimulator binding to the sGC heme domain of bacterial homologs, showing that binding at this site is conserved across species. The authors went further to characterize this heme binding domain, showing by MS and NMR data that the known stimulators bind to a conserved pocket lying between the two subdomains found in the heme domain of sGC. This work characterized and identified the specific location of binding, and thus permits better informed and structurally guided future design of NO stimulators.

**Figure 12:**
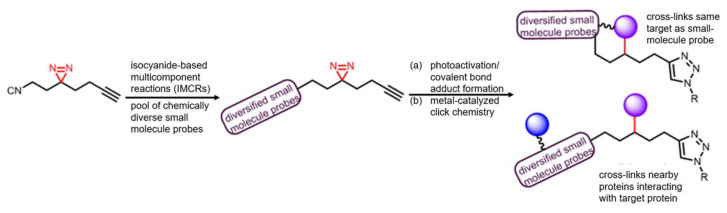
Bifunctional isocyanide probe facilitates multicomponent reactions and affords versatility and dual use as fragment-based screening and proteome mapping.

One possible limitation of the approaches described above is in the sheer size of the appendage groups, which may result in disruption of the probe’s function. Several methods thus require simple linkers that can facilitate similar studies with minimal disturbance of native activity. Jackson et al. [76] described a bifunctional isocyanide probe incorporating a DAZ group for target biomolecule capture and an aliphatic alkyne that allowed enrichment of photolabelled products via metal-catalyzed click chemistry. The isocyanide group permitted isocyanide-based multicomponent reactions (IMCR) that allowed installation of different small-molecule probes with diverse chemical scaffolds containing at least one aldehyde, amine, ketone, or carboxylic acid functionality (Figure 12). This approach thus introduced the added advantage of performing concurrent fragment-based screening and proteomic exploration under the same assay conditions. The outcome of this work was a clickable photoreactive isocyanide tool that is suited for combinatorial chemistry of IMCR fragments and can allow chemoproteomic-enabled drug discovery.

**Figure 13:**
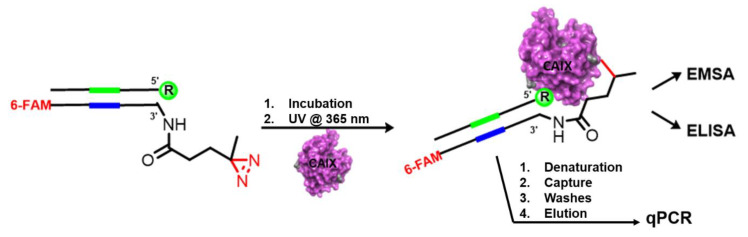
DAZ-functionalized oligonucleotide probes for studying protein–DNA interactions. (Electrophoretic mobility shift assay, enzyme-linked immunosorbent assay, and quantitative polymerase chain reaction are abbreviated to EMSA, ELISA, and qPCR respectively)

Several methodologies aim to improve the efficiency of screening procedures for identifying novel protein binders. DNA-encoded chemical libraries (DECLs) provide huge numbers of diverse chemical fragments that can be unambiguously screened against protein targets. The effectiveness of this approach is limited, as it involves several post-binding washes that restricts the retention and recovery of medium to low-affinity (K_d_) binders. One new approach that helps to uncover these micromolar binders with protein complexes that exhibit limited kinetic stability is via DAZ-mediated covalent crosslinking interactions. Following several reports [77,78] on the use of DAZ crosslinking reactions for DECL screenings, Sannino et al. [79] described a novel approach that involved a single-stranded oligonucleotide-encoded fragment library of which the complementary sequence carried a DAZ PCG. Using carbonic anhydrase IX (CAIX) as the model target protein, a combinatorial DECL was screened and irradiated at 365 nm to initiate covalent crosslinking of the DAZ with CAIX, the target protein, such that transient and low-affinity interactions were locked in (Figure 13). Following washes and elution steps, the authors were able to show that this crosslinking capture mechanism was able to identify low-affinity carbonic anhydrase IX binders when compared to traditional solid support-based affinity protein capture.

PL has been very instrumental in the structural characterization of specific binding sites, mainly for small-molecule probes. Complementary MS-enabled enrichment and NMR studies allow global proteomic studies that can spatiotemporally report these binding hotspots in an unbiased analytical procedure. The challenge, however, has been in extending this approach to larger MW and complex molecules; hence, the binding modes for these class of compounds have not been properly clarified.

Rapamycin is a 914 Da compound described as a molecular glue, as it stabilizes the FKBP12–mTOR protein complex. This ternary complex formation was previously described and characterized by biophysical methods such as crystallography [80]. In a bid to understand how PL can decode structural information on large-molecule binding locations, Flaxman et al. [81] designed photo-rapamycin by functionalizing the C40 (earlier shown to be amenable to functionalization) of rapamycin with a DAZ-containing bifunctional probe similar to that described by Jackson et al. [76]. Following incubation and photoirradiation of the photo-rapamycin, FKBP12, and mTOR, the authors showed formation of the ternary complex in vitro by MS fragmentation. They also showed that a McLafferty rearrangement of photo-rapamycin occurred at the ternary complex, and in conjunction with molecular dynamics studies, were able to characterize possible conformations of this ternary complex. This study thus further emphasized the utility of DAZ-based PCGs in PL studies.

#### 3.2.2. Studying Complex Protein Interactions

The use of PL techniques is not restricted to synthetic molecules alone. Mapping out complete protein interactomes is highly desirable for understanding cellular mechanisms and biological functions, and PL has been effectively used in elucidating some of these complex biomolecular interactions. Studies like these are highly relevant, as there are still a lot of unknown PPIs despite advancements in predictive computational tools and high-throughput mapping. These calculative techniques rely on pre-existing PPI databases for their algorithm-based predictions but are inadequate for elucidating novel PPIs with no prior knowledge of a relatable social protein network.

**Figure 14:**
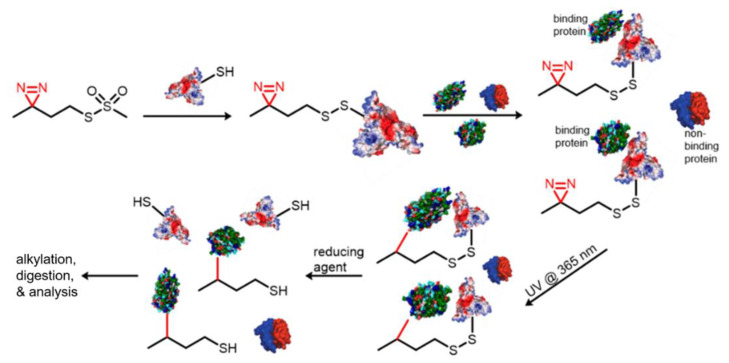
Bifunctional MTS-DAZ is a simple probe that can be used to investigate PPIs.

In a simple demonstration of how PL can be used to characterize new PPIs, Horne et al. [82] described the use of a DAZ-modified residue in a target protein that crosslinks interacting partners, and subsequent enrichment and MS characterization revealed these binding partners. In this approach, the authors introduced a reactive cysteine into the target protein to allow future incorporation of a disulfide bridge through a methanethiosulfonate (MTS) installed on the DAZ probe. On exposure to a pool of possible targets and subsequent irradiation of UV light at 365 nm, a crosslink was formed with binding proteins, treatment with reducing agents cleaved the disulfide bonds, and only the binding protein was retained (Figure 14).

Digestion and MS analysis revealed the identity of these interacting proteins. The authors believe that this strategy could potentially be used to map cellular PPIs in both precise and dynamic locations.

**Figure 15:**
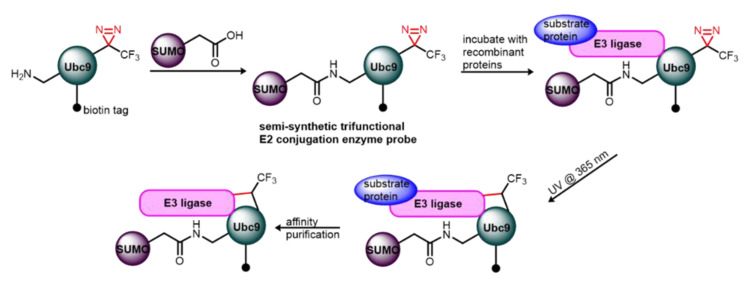
Trifunctional E2-conjugating enzyme probe to identify novel E3 ligase components of the SUMO-E2-E3 ternary complex.

A further extension of this approach was used to investigate the more complicated cellular protein machinery that regulates post-translational modifications such as SUMOylation, ubiquitination, and other ubiquitin-like (Ubl) modifications. The specific networking interactions of E1-activating and E2-conjugating enzymes combined with E3 ligases are modulated by several PTMs and cellular triggers that make their investigation somewhat challenging. Although the use of ABPP is a powerful tool that has been extensively used to study the specific interactomes and pathways of these PPIs [83,84,85,86,87], this method is limited to E3 ligases, which have a catalytic cysteine that is able to accept Ub/Ubl from the E2-conjugating enzyme and transfer them to the proteins present in their social network. The majority of E3 ligases, however, lack a reactive cysteine that can facilitate Ub/Ubl transfer and are reliant on transient non-covalent interactions to carry out their ubiquitination roles. To successfully investigate the vast abundance of Ub/Ubl-E2-E3 interactions that were excluded by the ABPP approach, Zhang et al. [88] constructed a trifunctional E2-conjugating enzyme probe that used an amide bond to link the SUMO-UBc9 (an E2-conjugating enzyme) system, which is known to have incredible affinity for E3 ligase. By functionalizing the Ubc9 with DAZ and photoactivation at 365 nm, any E3 ligase recruited is crosslinked covalently to form a SUMO-E2-E3 ternary complex (Figure 15). This approach should allow the validation of known E3 ligases and the discovery of novel E3 ligases hitherto unreported and should provide a strategy for the construction of other E2-Ub/Ubl conjugation frameworks.

**Figure 16:**
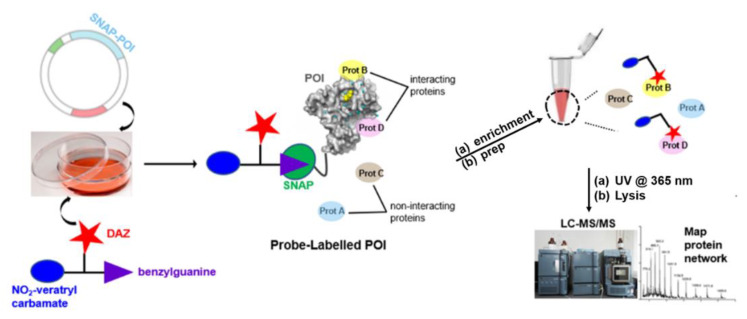
Workflow of photo-PPI that allows characterization of a protein’s social network in live cells.

The utility of DAZ-based PL techniques has been extended into biological studies of protein interactions in live cells, where there is a myriad of highly complex, interconnected series of molecular interaction networks occurring concurrently. The resolution and identification of these interactions for a specific biomolecule of interest with no or minimal perturbation of these cellular events is highly challenging. However, this ability increases our understanding of how cellular events are organized in time and space and provides insight on how to effectively modulate these events, especially for therapeutic purposes. Current techniques employed to study these myriad interactions include affinity purification–mass spectroscopy (AP-MS), chemical/enzymatic-tagging-based LC-MS/MS, BioID, and proximal enzymatic labelling [89,90,91,92,93,94]. While some of these techniques have been successful, they also come with some significant limitations such as bias against weaker interactions, need for co-factors, and labelling approaches that require initiation by exogenous chemicals which have the potential to unsettle normal cellular homeostasis [95,96]. McCutcheon et al. [97] reported a photo-proximity PPI (PhotoPPI) profiling method that allowed the characterization of PPI networking maps in vitro and in live cells. The authors employed a SNAP-tagged protein of interest (POI) and functionalized their DAZ-based probe with a benzylguanine and a photocleavable group (NO_2_-veratryl carbamate), such that the DAZ-functionalized probe was covalently attached to the POI (e.g., redox regulated sensor protein, KEAP1) by the benzylguanine moiety. Subsequently, upon irradiation at 365 nm, the NO_2_-veratryl-carbamate group was cleaved off and any proximal proteins to the POI were simultaneously labelled by the DAZ group, thereby identifying any nearby proteins with the POI via a subsequent enrichment and quantitative LC-MS/MS analysis (Figure 16).

This technique was used by the authors to elucidate the interactome for KEAP1, validate known interactions, and discover novel ones for KEAP1 with high temporal resolution in live cells [97].

#### 3.2.3. Probing Membrane Proteins and Other Protein–Biomolecule Interactions

**Figure 17:**
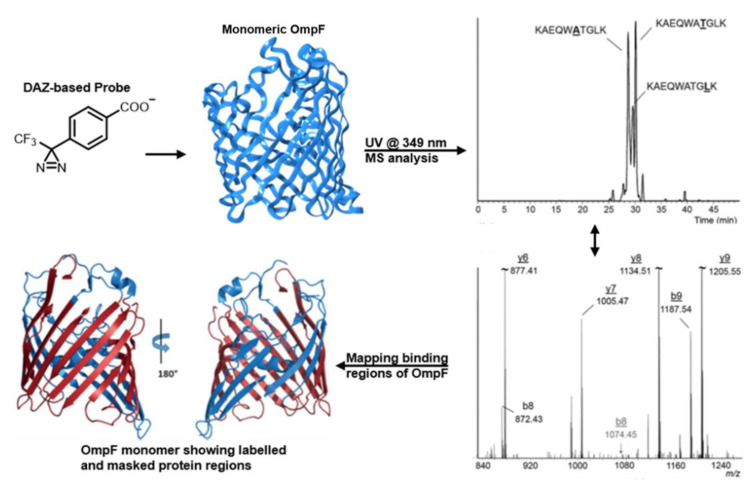
DAZ-modified OmpF allows successful mapping of proteins’ binding regions. Protein in red indicates regions that were successfully labelled by the probe; protein in blue indicates unlabelled (masked) regions of the protein. (Image showing labelled and masked protein region adapted with permission from Manzi et al., 2017 [107]).

DAZ-based PL is a highly versatile technique that has been applied to the study of several biomolecular interactions and processes. DAZ-based PL was recently applied to the study of membrane proteins, a very challenging field of study in chemical biology. Despite the important roles that membrane proteins play in critical cellular processes such as cellular transport, signaling, and reception, their structures and mechanisms are still poorly understood. Some of the current techniques applied to study them include X-ray crystallography [98,99,100], cryo-EM [100], solid-state NMR [101,102], and MS [103,104,105,106]. Studying membrane proteins using X-ray crystallography has been a tricky approach due to the hydrophobic nature of these proteins, which increases the likelihood that they will aggregate and precipitate out of solution. Cryo-EM and solid-state NMR, on the other hand, have shown a lot of promise in this regard, but MS is arguably the most effective method to date for studying membrane proteins. However, the number of techniques available to study membrane proteins is still small when compared to their soluble counterparts, underscoring the need for new techniques that can provide valuable information on this class of proteins. In a proof-of-concept design, Manzi et al. [107] described a DAZ-based label (Figure 17) that was able to efficiently label the β-barrel porin protein OmpF, which is an outer membrane protein responsible for small-molecule diffusion in *E. coli*. They were able to show that their probe, due to its amphipathic nature, could access the micelle formed by OmpF and discriminately label and distinguish the hydrophobic transmembrane sections, as opposed to the subunit interfacial binding sections. By using MS/MS analysis, mapping of the surface-accessible binding sections in OmpF was possible.

There is a paucity of techniques available for studying protein–lipid interactions. Lipids are key building blocks in cells, involved in protein lipidation, modulate subcellular transport, and are major components of cellular membranes. The protein–lipid interaction is a key metabolic interaction that is responsible for a host of cellular signaling and function and designing techniques that can be used to investigate these interactions is highly desirable. The use of DAZ-based PL has been applied to the study of lipid–protein interactions by some research groups [108,109], but, because of the need to chemically synthesize and incorporate their probes into the lipid design, they could not efficiently replicate innate cellular metabolic strategies using solely biologically synthesized lipids. In 2017, Wang et al. [110] reported a semi-synthetic bifunctional probe design that could be incorporated into cellular phospholipid biosynthesis and used this strategy to identify many binding protein partners to the phospholipid phosphatidylcholine.

**Figure 18:**
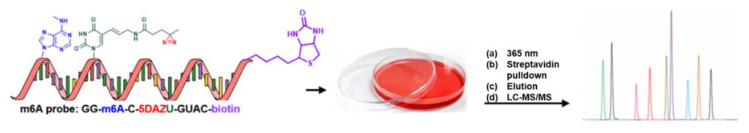
DAZ-modified RNA close to established m6a RNA modifications allows elucidation of binding partners that recognize this modification.

Another complex protein interaction that has been challenging to efficiently characterize is the protein–RNA interaction, due to the low affinity of these interactions, and the presence of accompanying protein–protein complexes usually complicates these studies. Protein–RNA interactions help to mediate cellular control of RNA localization, stability, splicing, and translation. The N6-methyladenosine (m^6^A) RNA post-transcriptional modification is a newly identified RNA modification that is postulated to regulate gene expression via subjective recognition by specific proteins. In a study to identify protein partners that recognize the m^6^A RNA modification, Arguello et al. [111] described an RNA photoaffinity probe that contained the m^6^A modification adjacent to a non-perturbing DAZ PCG, and a biotin affinity handle for subsequent protein enrichment post-crosslinking. This RNA probe was introduced into HeLa cell lysate, and irradiation at 365 nm ensured crosslinking to interacting proteins. Streptavidin enrichment and LC-MS/MS analysis were used to identify and quantify protein interacting with the m^6^A RNA modification and their abundance (Figure 18). This study further established the utility of DAZ probes in mapping biological interactomes, and the probe designed here should be applicable to the investigation of interacting partners of any post-transcriptional RNA modification.

DAZ-based techniques have also been applied to immobilization of biomolecules on solid support systems to enable straightforward and selective capture of biochemical materials of interest. Multifunctional nanoparticles (MNPs) such as supra-paramagnetic iron oxides have been highly successful in surface chemistry applications for the controlled and site-specific immobilization of biological species facilitated by amide bonding [112]. This strategy can sometimes jeopardize the biological function of immobilized species, especially antibodies (Abs), due to blockage of their binding sites [113]. Other conjugation protocols such as thiol reduction or oxidation also fall short, due to harsh chemical treatments that may alter Ab structure and function. Fan et al. [113] reported an MNP functionalized with a boronic acid for Ab capture and a DAZ to efficiently crosslink and covalently immobilize the Ab to the metal surface. They also showed that this approach was a superior immobilization strategy compared to other known approaches. The immobilized Ab maintained excellent stability and an active orientation that allowed selective binding for the target protein. These studies continue to emphasize the utility of DAZ-based PL probes and the variations in their design that make them applicable to various biological investigations.

#### 3.2.4. Molecular Markers for Imaging Studies

Imaging techniques such as NMR and MRI represent powerful analytical tools that have found widespread use in academic investigation and clinical diagnosis, respectively. The biggest pitfall of both applications is in their low sensitivities arising from their low nuclear spin polarization, a feature that describes the population of nuclear spins that can transit different energy levels. High-field NMR/MRI [114], aside from other challenges, cannot considerably increase nuclear spin polarization to the level that will significantly enhance technique sensitivity. The use of hyperpolarizing agents is one way to dramatically increase signal intensities and improve sensitivity. Some examples include dynamic nuclear polarization (DNP) [115], hyperpolarized gases [116,117], parahydrogen-induced polarization [118], and signal amplification by reversible exchange (SABRE) [119,120].

**Figure 19:**
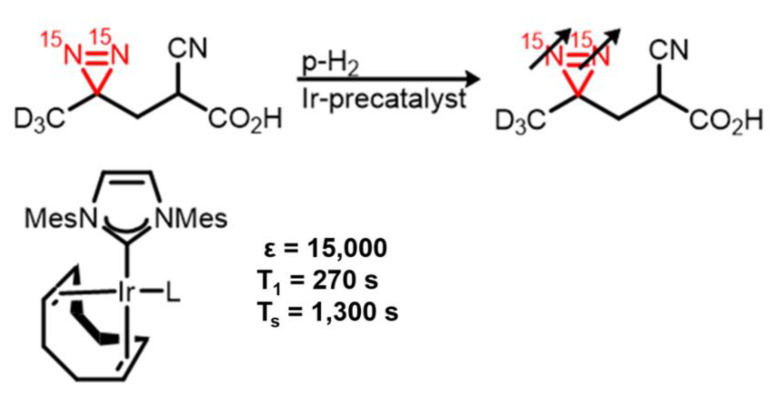
The use of DAZ as hyperpolarized markers for SABRE leads to significant enhancement in signals.

Hyperpolarization by SABRE improves nuclear spin polarization via rapid and reversible exchange of parahydrogen (p-H_2_) with the available sites of a metal complex (usually iridium, Ir), and at the microtesla (µT) magnetic field strength usually employed, p-H_2_ spin polarization transfer is possible all the way to substrate molecules such as ^15^N-pyridine. This technique has been shown to provide significant gain in signal intensities that can translate to ^15^N biomedical imaging and spectroscopy [121,122,123]. ^15^N-DAZ has been proposed as an alternative to ^15^N-pyridines as substrate for SABRE technique due to its small size, ease of incorporation, longer polarization lifetime, and non-perturbance of native biomolecule activity [124]. The potential of ^15^N-DAZ as an imaging tag was investigated by Shen et al. [124], showing a 15,000-fold signal enhancement (ε) over ^14^N-DAZ (Figure 19). They also found several-fold increases in the singlet spin order (T_s_) and magnetization relaxation (T_1_) time constants of ^15^N_2_ when compared to normal polarization decay time constants. On incorporation of their ^15^N-DAZ into choline derivatives, they observed a 2000-fold increase in signal with T_1_ = 3 min. Their set-up also displayed extended polarization lifetimes and signal enhancement in D_2_O, suggesting the applicability of this tag for in vivo biomedical imaging applications. In a subsequent follow-up paper [125], the same authors described the design of new terminal ^15^N-DAZ-based tags that are easier to incorporate, can substitute for methyl groups in biological structures, and display similar relaxation time constants as the earlier tag (Figure 19), validating the robustness of this hyperpolarized imaging tag.

Synthetic methods to introduce terminal DAZ into biomolecules are of interest as functionalization at this position should impose the least disturbance on activity. The installation of terminal DAZ is, however, poorly exploited. Glachet et al. [54] showed a one-pot three-step metal-free synthetic procedure for the synthesis of terminal DAZ directly from amino acids. The scope of this reaction was evaluated in several natural and unnatural amino acids, showing good yields in most cases. These reaction scopes provide a template for future functionalization of peptides and proteins.

In a validation of the applicability of a ^15^N-DAZ-based platform for hyperpolarization studies, Park et al. [126] constructed an array of several ^15^N-DAZ-tagged biomolecules ranging from simple sugars and amino acids to drug molecules. The authors initially used SABRE to demonstrate the feasibility of ^15^N-DAZ hyperpolarization which, despite showing excellent signal enhancement and extended hyperpolarized lifetimes, could not be carried forward as method of choice due to its preference for organic solvents over aqueous solutions. In this new study, the authors decided to use DNP as an alternative approach due to its versatility and broad applicability.

They functionalized known molecules such as glucose, the drug colecoxib, and several amino acids with ^15^N-DAZ and were able to show signal enhancements up to 400,000-fold and relaxation times T_1_ of up to 4 min. These experiments were also conducted in D_2_O, suggesting the potential of the technique for application in clinical imaging research. These works highlight the promising potential of ^15^N-DAZ molecular tags as future hyperpolarized imaging agents for use in clinical diagnostics and biological research.

## 4. Future Directions

BPs and DAZs have become two mainstay PCGs that are used today in PL studies due to intrinsic properties that make them amenable for use in studies of biological systems. DAZs such as TPD have gained significant interest because of their chemical stability and small size, which is expected to minimally alter functions of the system of study. However, the chemical compatibility of DAZs to reaction conditions limits the point of incorporation in the synthesis as well as the position on the probe. Additionally, the study performed by Ichishi et al. optimizing the reaction conditions for a Suzkui–Miyaura compatible with the TPD system [50] will enable the future application of biphenyl DAZ-labelled motifs, a commonly used motif in medicinal chemistry, to the study of drug–target interactions. Further studies aimed at the expansion of the synthetic compatibility of DAZs with common synthetic reactions may enable the incorporation of DAZs with minimal perturbation (nested DAZs, Figure 6c) to the chemical system being studied. Moreover, the usual synthetic methods to synthesize DAZs are inefficient and low yielding, and attempts to discover more convenient and efficient routes, such as those discovered by Wang et al. [52,53] and Glachet et al. [54] will likely make DAZs attractive PCGs for PL and other applications.

The utility of both classes of PCG has enabled their incorporation into traditional PL approaches in elucidating diverse biochemical interactions of synthetic and biological micro and macro molecules in both in vitro and in cellulo studies. The use of both BPs and DAZs in solid-support immobilization systems continues to increase, and more studies that integrates them as covalent conjugation systems are expected. Simple and versatile designs such as IMCR, the *ortho*-Nosyl-DAZ probe, and the SUMO-E2-DAZ probe allow multicomponent studies and effectively use DAZ for light-controlled crosslinking, and their further development, validation, and widespread use are expected. These tools allow multitarget installation such that several concurrent studies can take place in the same system. They can be used to study ternary and quaternary biological interactions, and can integrate drug screening, target identification, and proteomic studies into a single probe. Because these designs are simple and easy to install and modify, their adaptation to other multicomponent system studies should be anticipated. Imaging systems that rely on DAZs as hyperpolarizing agents continue to gain significant attention and this is expected to continue. The advancement of DAZ agents into signal enhancers and contrasting agents in NMR investigations and MRI diagnosis should allow for the combination of these diagnostic tools with proteomic studies that already use DAZ probes. DAZ and BP probes thus hold substantial promise as a one-system multifunctional tool to synchronously investigate and diagnose a myriad of biological processes.
